# Chromosomal Differentiation of *Deschampsia* (Poaceae) Based on Four Satellite DNA Families

**DOI:** 10.3389/fgene.2021.728664

**Published:** 2021-09-21

**Authors:** María Laura González, Jorge Oscar Chiapella, Juan Domingo Urdampilleta

**Affiliations:** ^1^ Instituto Multidisciplinario de Biología Vegetal (Consejo Nacional de Investigaciones Científicas y Técnicas - Universidad Nacional de Córdoba), Córdoba, Argentina; ^2^ Instituto de Investigaciones en Biodiversidad y Medioambiente (Consejo Nacional de Investigaciones Científicas y Técnicas - Universidad Nacional Del Comahue), Bariloche, Argentina

**Keywords:** Deschampsia, cytogenetics, repetitive DNA, satellite DNA, FISH

## Abstract

Diverse families of satellite DNA (satDNA) were detected in heterochromatin regions of *Deschampsia*. This kind of repetitive DNA consists of tandem repeat sequences forming big arrays in genomes, and can contribute to lineages differentiation. The differentiation between types of satDNA is related to their sequence identity, the size and number of monomers forming the array, and their chromosomal location. In this work, four families of satDNA (D2, D3, D12, D13), previously isolated by genomic analysis, were studied on chromosomal preparations of 12 species of *Deschampsia* (*D. airiformis*, *D. antarctica*, *D. cespitosa*, *D. cordillerarum*, *D. elongata*, *D. kingii*, *D. laxa*, *D. mendocina*, *D. parvula*, *D. patula*, *D. venustula,* and *Deschampsia sp*) and one of *Deyeuxia* (*D. eminens*). Despite the number of satDNA loci showing interspecific variation, the general distribution pattern of each satDNA family is maintained. The four satDNA families are AT-rich and associated with DAPI + heterochromatin regions. D2, D3, and D12 have mainly subterminal distribution, while D13 is distributed in intercalary regions. Such conservation of satDNA patterns suggests a not random distribution in genomes, where the variation between species is mainly associated with the array size and the loci number. The presence of satDNA in all species studied suggests a low genetic differentiation of sequences. On the other hand, the variation of the distribution pattern of satDNA has no clear association with phylogeny. This may be related to high differential amplification and contraction of sequences between lineages, as explained by the library model.

## Introduction

Repetitive DNA is one of the main components of plant genomes, reaching about 50–90% of genomic abundance (Bennett et al., 1982; Kubis et al., 1998; Heslop-Harrison, 2000), and is composed by dispersed (e.g., transposable elements) and tandem (e.g., satellite DNA) sequences, with variable abundance. Since satellite DNA (satDNA) usually form large arrays on chromosomes (frequently related to heterochromatin regions), it is detectable by fluorescence *in situ* hybridization (FISH) (Schwarzacher and Heslop-Harrison, 2000), allowing their study at the cytogenetical level. The main characteristics defining satDNA are monomer size over 100–150 bp and arrays up to 100 Mb with tandem disposition (Sharma and Raina, 2005; Hemleben et al., 2007; Mehrotra and Goyal, 2014). Although they are considered non-coding sequences, their monomeric size frequently varies between 150–180 and 320–360 bp, which corresponds with the structural motifs of mono- and dinucleosomes (Kubis et al., 1998; Macas et al., 2002). The current genomic sequencing methods (e.g., NGS), together with the advance of bioinformatics, constantly provide new data about the structural diversity of satDNA (Ruiz-Ruano et al., 2016; Garrido-Ramos, 2017; Lower et al., 2018), however many aspects remain to be understood.

The evolution of satDNA in genomes implies that both sequences diverge as changes in copy numbers. The abundance of satDNA in eukaryote genomes can vary widely and rapidly between generations, leading to high polymorphism in the satellite arrays’ length (Plohl et al., 2012). The sequence identity inside an array evolves according to the process called concerted evolution, which causes more similar monomers than expected due to random changes (Dover, 1986; Plohl, 2010). The monomers homogenization is much faster in species with sexual reproduction, given meiotic recombination (Mantovani et al., 1997). The chromosome organization has a fundamental influence on processes such as chromosome pairing, segregation, gene organization, and expression, and repetitive DNA such as satDNA have an important role in DNA packaging and chromatin condensation (Heslop-Harrison, 2000). Since satDNA distribution may facilitate the recognition of homologous chromosome pairs, changes between lineages have been precursors of speciation (Hemleben et al., 2007). The similarities and differences in genomic satDNA between species can be explained by the “library model”, which suggests differential amplification of satDNA between independent lineages (Garrido-Ramos, 2015). However, the patchy distribution of some satDNA types across eukaryotes (animal and plants) suggests that a scenario of multiple horizontal transfers during evolution may be considered (Yang et al., 2020).

The grass genus *Deschampsia* P. Beauv. is a cosmopolitan genus which includes about 30 species, 15 of them growing in South America, including *D. antarctica* which also occurs in Antarctica (Parodi, 1949; Chiapella and Zuloaga, 2010). Some species have difficult circumscription, forming species complexes (Chiapella et al., 2011; Tzvelev et al., 2015). Regarding *Deschampsia* cytogenetics, the species show a basic chromosome number of x = 13, with a few exceptions reported in the northern hemisphere. The most common chromosome number is 2n = 26, followed by 2n = 52, and the chromosomal complement has metacentric, submetacentric, and acrocentric chromosomes, in similar proportions between species (Albers, 1980; Winterfeld and Röser, 2007b; Cardone et al., 2009; González et al., 2021). In contrast with the conserved chromosome morphology, the *18-5.8-26S* and *5S* rDNA patterns have high intra and inter-specific variability of loci number and position, which allow the determination of chromosome markers specific to some phylogenetically related species groups (Chiapella, 2007; Saarela et al., 2017; González et al., 2021).

Several satDNA families were isolated from tribe Poeae using restriction enzymes (Grebenstein et al., 1995, 1996), and some of them are present in various genera, including *Deschampsia*, suggesting an ancient origin (Röser et al., 2014). Likewise, the occurrence of several specific satDNA types were reported in different groups of grasses (Anamthawat-Jónsson and Heslop-Harrison, 1993; Vershinin et al., 1995; Nakajima et al., 1996). The analysis of repetitive DNA from WGS in *D. antarctica* and *D. cespitosa* allowed researchers to recognize 34 satDNA families (González et al., 2020). This is a high satDNA diversity compared to other grasses, such as *Eragrostis tef* (one satDNA family), *Agropyron cristatum* (fourteen satDNA families), *Festuca pratensis* Huds. (eight satDNA families), and *Poa* (four satDNA families) (Gebre et al., 2016; Křivánková et al., 2017; Said et al., 2018; Wei et al., 2020)*. Deschampsia antarctica* and *D. cespitosa* showed a slightly differentiated satDNA genomic pattern when considering abundance and diversity. Two types of chromosome distribution patterns were observed in the analyzed satDNA families, a C-type pattern (clustered) and M-type pattern (mixed combination of both clustered and dispersed) (González et al., 2020).

The use of C-type satDNA in *Deschampsia* would allow us to reveal if there exists interspecific variation of the distribution of such sequences in chromosomes, which may be related to the speciation processes. In this way, D2 (359 pb), D3 (377 pb), D12 (366 pb), and D13 (563 pb) families previously analyzed in *D. antarctica* and *D. cespitosa* (González et al., 2020), could be good markers to analyze the interspecific variation. The phylogenetic relationships between the South American species were inferred with molecular data and suggest a recent common ancestor between *Deschampsia* and *Deyeuxia* sec. *Stylagrostis*, which could have had chromosomal characteristics currently shared by both taxa (Saarela et al., 2017; González et al., 2021). Due to phylogenetic results, Saarela et al. (2017) suggested transferring seven South American species of *Deyeuxia* sect. *Stylagrostis* to *Deschampsia*, hence more studies of new sources of variation will be useful. With the aim of evaluating karyotypic variations related to phylogenetic hypotheses we analyzed chromosomal distribution of some satDNA families in twelve different *Deschampsia* species and one of *Deyeuxia eminens.*

## Material and Methods

### Plant Material

We collected twelve species of *Deschampsia* (one species, vouchers MLG 79 and MLG 81, does not match any described species), *Deyeuxia eminens* (which belongs to sect. *Stylagrostis*), and *Avenella flexuosa* from Antarctica and Argentinian Patagonia (Table 1). Living plants were transported to the laboratory and kept in pots in culture chambers at 14°C, to obtain root tips for cytogenetic techniques. Leaves were kept in silica gel for DNA extractions. The vouchers were included in the collection of the herbarium of the Botanical Museum of Córdoba (CORD).

**TABLE 1:** Studied species and results from FISH with satDNA. CORD: herbarium number (MLG: Maria Laura González; JC: Jorge Chiapella; JDU: Juan Domingo Urdampilleta; MG: Melisa Giorgis). 2n: somatic chromosome number. N: number of hybridization signals. P: hybridization pattern (st: subterminal, i: intercalary, *m*: metacentric, *sm*: submetacentric, *a*: acrocentric). Arg: *Argentina*.

Species	CORD	Locality	2n	D2		D3		D12		D13	
				N	P	N	P	N	P	N	P
*D. airiformis*	MLG 41	Arg, Chubut, Tehuelches	26	6	4 st *m*; 2 st *a*	4	2 st *m*; 2 st *a*	–	–	–	–
*D. antarctica*	JC 2775	Antarctic, 25 de Mayo Island	26	8	6 st *m*; 2 i *a*	12	10 st *m*; 4 st *a*	4	2–6 st *m*; 0–2 st *a*	2	2 i *a*
*D. cespitosa*	MLG 35	Arg, Chubut, Río Senguer	26	6	6 st *m*	7	6 st *m*; 1 st *sm*	4	4 st *m*	2	2 i *a*
	JDU 850	Arg, Chubut, Rio Senger	26	6	4 st *m*; 2 i *a*	–	–	–	–	–	–
	JDU 823	Arg, Mendoza, Malargüe	26	8	5 st *m*; 2 st *sm*; 1 st *a*	7	7 st *m*	–	–	–	–
*D. cordillerarum*	MLG 69	Arg, Mendoza, Las Heras	26	11	3 st *m*; 2 st *sm*; 2 i *a*; 4 st *a*	12	6 st *m*; 2 st *sm*; 4 st *a*	–	–	–	2 i *a*
*D. elongata*	MLG 56	Arg, Chubut, Languiñeo	26	12	8 st *m*; 4 i *a*	8	8 st *m*	10	8 st *m*; 2 i *sm*	2	2 i *a*
*D. kingii*	JDU 842	Arg, Chubut, Languiñeo	52	10	8 st *m*; 2 st *a*	20	18 st *m*; 2 st *a*	6	4 st *m*; 2 st *a*	2	2 i *a*
*D. laxa*	MLG 114	Arg, Rio Negro, Bariloche	26	10	7 st *m*; 2 i *a*: 1 i/st *a*	7	7 st *m*	4	4 st *m*	2	2 i *a*
*D. mendocina*	MLG 91	Arg, Mendoza, Malargüe	26	12	6 st *m*; 2 st *sm*; 2 i *a*; 2 st *a*	12	8 st *m*; 2 st *sm*; 2 st *a*	–	–	–	–
	MLG 101	Arg, Mendoza, Malargüe	26	–	–	–	–	4	4 st *m*	2	2 i *a*
*D. parvula*	MLG 48	Arg, Chubut, Languiñeo	26	6	4 st *m*; 2 i *a*	8	4 st *m*; 2 st *sm*; 2 st *a*	6	4 st *m*; 2 st *a*	2	2 i *a*
*D. patula*	JDU 878	Arg, Santa Cruz, Güer Aike	26	6	4 st *m*; 2 i *a*	10	8 st *m*; 2 st *a*	–	–	–	–
*D. venustula*	MLG 62	Arg, Neuquén, Picunches	26	6	2 st *m*; 4 i *a*	4	2 st *m*; 2 st *sm*	4	4 st *m*	2	2 i *a*
*Deschampsia* sp	MLG 81	Arg, Mendoza, San Carlos	26	21	10 st *m*; 2 st *sm*; 4 i *a*; 5 st *a*	15	10 st *m*; 2 st *sm*; 3 st *a*	3	3 st *m*	2	2 i *a*
*Deyeuxia eminens*	MG 1835	Arg, Córdoba, Calamuchita	26	9	1 st *m*; 1 st *sm*; 4 i *a*; 1 st *a*; 1 i/st *a*	11	5 st *m*; 2 st *sm*; 4 st *a*	–	–	–	–
*A. flexuosa*	JDU 848	Arg, Chubut, Languiñeo	28	0	–	0	–	–	–	–	–

### Cytogenetic Techniques

The mitotic chromosomes preparations were obtained from root meristems pretreated with 2 mM 8-hydroxyquinoline for 4–6 h at 14°C and fixed in ethanol/acetic acid (3:1, v:v). The tissues were digested with Pectinex enzyme solution (Novozimes) and squashed in 45% acetic acid. Preparations were frozen in liquid nitrogen to remove the coverslip.

Fluorescence *in situ* hybridization (FISH) was carried out to detect satellite DNA patterns, following protocols by Schwarzacher and Heslop-Harrison (2000). To reach stringency above 76%, the hybridization mix had 2x SSC, 50% v/v Formamide, 20% v/v dextran sulfate, 0.1% v/v SDS, and 4–6 ng/μL probes. Post-hybridization washes consisted of 2x SSC, 0.1x SSC, 2× SSC, for 10 min at 42 °C each. We selected four families with a conspicuous hybridization pattern among the previously isolated and mapped satDNA families in *D. antarctica* and *D. cespitosa* to analyze chromosomal distribution in other *Deschampsia* species and related taxa: D2, D3, D12, and D13. Probes were obtained by PCR as described in González et al. (2020) and labelled with biotin (Bionick, Invitrogen) (D2 and D13) or digoxigenin (D3 and D12) (DIG Nick translation mix, Roche). Double-target FISH was performed by hybridizing D2 with D3, and D12 with D13. The satDNA D2 and D3 were mapped in 14 species: *D.* cfr. *airiformis*, *D. antarctica*, *D. cespitosa* (three localities were used), *D. cordillerarum*, *D. elongata*, *D. kingii*, *D.* cfr. *laxa*, *D.* cfr. *mendocina*, *D. parvula*, *D. patula*, *D. venustula*, *Deschampsia* sp, *A. flexuosa,* and *Deyeuxia eminens*. The satDNA D12 and D13 were mapped in nine species: *D. antarctica*, *D. cespitosa*, *D. elongata*, *D. kingii*, *D. laxa*, *D. mendocina*, *D. parvula*, *D. venustula*, and *Deschampsia sp*. The detection was made with Avidin-FITC (Sigma-Aldrich) and anti-DIG antibodies conjugated with rhodamine (Roche). Chromosome metaphases were photographed using an Olympus BX61 microscope coupled with a monochromatic camera and Cytovision software (Leica Biosystems), and the images were pseudo-colored. Idiograms were constructed for *Deschampsia* species and *Deyeuxia eminens*. The karyotype conformation of each species was taken from González et al. (2021).

### Reconstruction of Ancestral States of Chromosomal Traits

For the reconstruction of ancestral states of satDNA chromosomal traits, the packages *ape* (Paradis et al., 2004), *geiger* (Harmon et al., 2008), and *phytools* (Revell, 2012) were used in R. The phylogenetic hypothesis used was the maximum likelihood tree reconstructed with *ITS*, *ETS,* and *trn*L-F markers by González et al. (2021), which includes the same plant vouchers used for the cytogenetic analysis of this study. The ER (equal transition rates) and ARD (all transition rates are different) models were tested for discrete traits. For continuous traits, the models BM (Brownian motion), OU (Ornstein-Uhlenbeck), and EB (Early-burst) were tested. The models were fitted using the package *geiger*, and the best models were selected according to AIC criterion for each trait. The reconstruction of ancestral states of discrete traits was carried out using maximum likelihood, with the Markov k-state (Mk1) model according to the better-adjusted transition model using the package *ape*. The reconstruction of ancestral states of continuous traits was carried out using the package *phytools*. The phylogenetic signal was tested using the package *geiger*.

## Results

**FIGURE 1:**
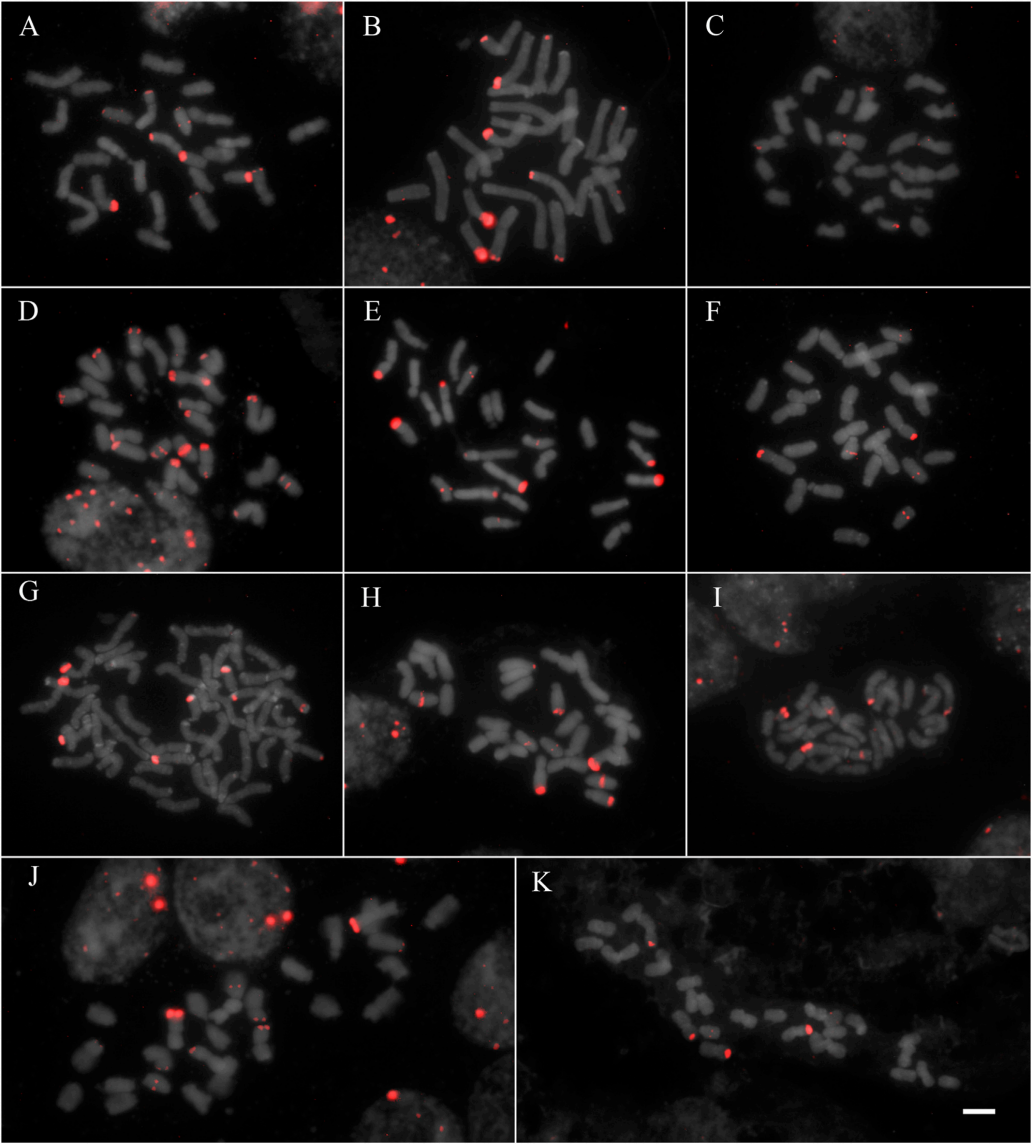
FISH of satDNA D2 (red) of studied species **(A)** *D. cordillerarum* MLG 69 **(B)** *D. mendocina* MLG 91 **(C)** *D. airiformis* MLG 41 **(D)** *Deschampsia* sp MLG 81 **(E)** *D. elongata* MLG 56 **(F)** *D. venustula* MLG 62 **(G)** *D. kingii* JDU 842 **(H)** *Deyeuxia eminens* MG 1835 **(I)** *D. patula* 878 **(J)** *D. laxa* MLG 114 **(K)** *D. parvula* MLG 48. Scale: 5 µm.

**FIGURE 2:**
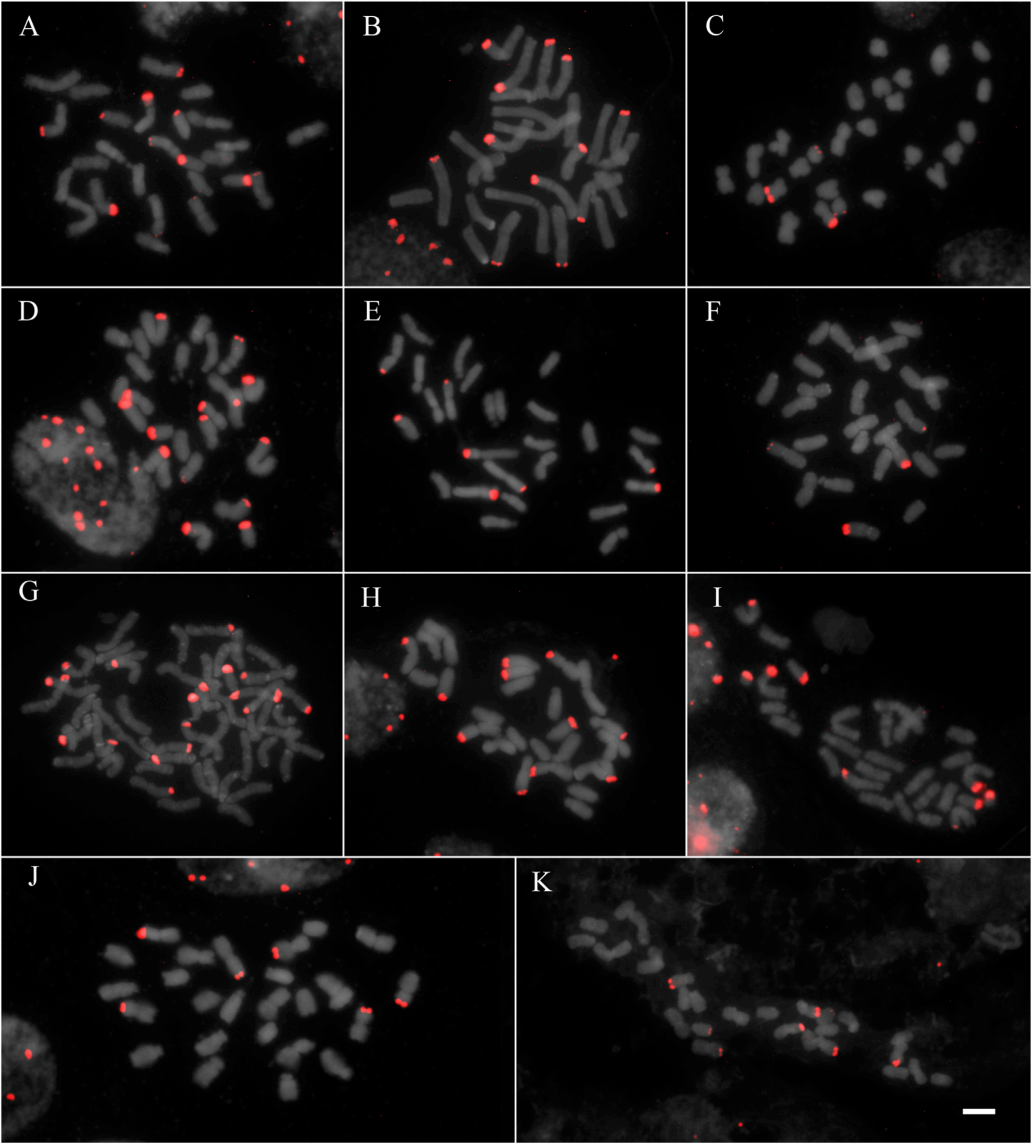
FISH of satDNA D3 (red) of studied species **(A)** *D. cordillerarum* MLG 69 **(B)** *D. mendocina* MLG 91 **(C)** *D. airiformis* MLG 41 **(D)** *Deschampsia* sp MLG 81 **(E)** *D. elongata* MLG 56 **(F)** *D. venustula* MLG 62 **(G)** *D. kingii* JDU 842 **(H)** *Deyeuxia eminens* MG 1835 **(I)** *D. patula* 878 **(J)** *D. laxa* MLG 114 **(K)** *D. parvula* MLG 48. Scale: 5 µm.

**FIGURE 3:**
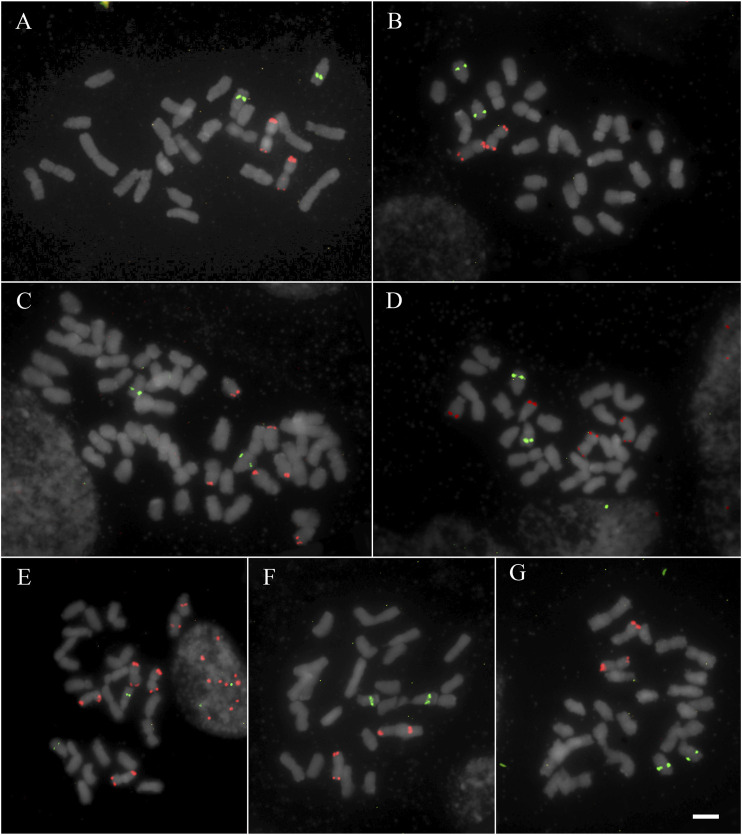
FISH of satDNAs D12 (red) and D13 (green) of studied species **(A)** *D. venustula* MLG 62 **(B)** *D. laxa* MLG 114 **(C)** *D. kingii* JDU 842 **(D)** *D. parvula* MLG 48 **(E)** *D. elongata* MLG 56 **(F)** *D. mendocina* MLG 101 **(G)** *Deschampsia* sp MLG 81. Scale: 5 µm.

All studied species showed hybridization to all studied probes, except for *A. flexuosa* which did not show hybridization for D2 and D3 satDNA probes (Table 1). The four satDNA families studied here are AT-rich and were frequently observed in association with DAPI + bands observed after FISH (Supplementary Figure S1, S2, S3, and S4). The species showed variation of the number and position of loci. The satDNA D2 showed variation of the loci number, ranging from 6 to 21 hybridization signals between species (which corresponded from 2.5 to 10.5 signals per basic complement x), noting that most species showed six hybridization signals (Figure 1; Table 1). The satDNA D3 showed from 4 to 20 hybridization signals (from 2 to 7.5 per basic complement x) (Figure 2; Table 1). The satDNA D12 showed from 3 to 10 hybridization signals between species (from 1.5 to five per basic complement x), noting that most species showed four hybridization signals (Figure 3; Table 1). The satDNA D13 was constant in all studied species, showing 2 hybridization signals (corresponding with 0.5–1 locus per basic complement x, since there was one tetraploid species) (Figure 3; Table 1). Only considering D2 and D3, since they were hybridized in all species, the species with more satDNA loci per basic complement was *Deschampsia sp*, followed by *D. mendocina* and *D. cordillerarum* (Table 1). Likewise, *D. elongata* was the species with the highest number of signals observed for D12.

The satDNA family D2 was mostly located at subterminal regions of different chromosome types, forming blocks of variable size. All species showed subterminal loci of D2 on metacentric chromosomes, and the species group formed by *D. cordillerarum*, *D. mendocina*, *Deyeuxia eminens,* and *Deschampsia sp* showed subterminal loci on submetacentric chromosomes. Also, some loci were found in acrocentric chromosomes, at an intercalary position in most species (except for *D. kingii* and *D. airiformis*) and at a subterminal position for *D. cordillerarum*, *D. mendocina*, *Deyeuxia eminens*, *Deschampsia sp*, *D. airiformis,* and *D. kingii* (Figure 1 and Supplementary Figure S4).

The satDNA family D3 was exclusively observed at subterminal regions. All species showed D3 loci on metacentric chromosomes, and most species also showed on the long arm of submetacentric chromosomes except for *D. laxa*, *D. airiformis*, *D. elongata*, *D. antarctica,* and *D. patula*. The satDNA D3 was also observed on acrocentric chromosomes at a subterminal position in most species, except for *D. cespitosa*, *D. laxa*, *D. venustula,* and *D. elongata.* This satDNA was frequently co-localized with D2 (Figures 1, 2 and Supplementary Figure S1, S2, S3, and S4).

The satDNA family D12 was detected at a subterminal position on metacentric chromosomes in all studied species. Particularly for D12, most of the studied species showed metacentric chromosome pairs with hybridization signals in both arms (*symmetric chromosome*). In general, the species showed only one chromosome pair with satDNA D12 (in one or two chromosomal arms), except for *D. kingii*, *D. antarctica,* and *D. elongata*, which showed three or four pairs. This satDNA was also detected in acrocentric chromosomes at a subterminal position in *D. antarctica*, *D. parvula,* and *D. kingii. Deschampsia elongata* was the only species showing one locus of D12 on submetacentric chromosomes (Figure 3 and Supplementary Figure S4).

The chromosomal distribution of satDNA family D13 was conserved in the analyzed species (Figure 3 and Supplementary Figure S4). We observed only one locus at an intercalary position on an acrocentric chromosome pair for all the species, inclusive for the tetraploid *D. kingii*.

**FIGURE 4:**
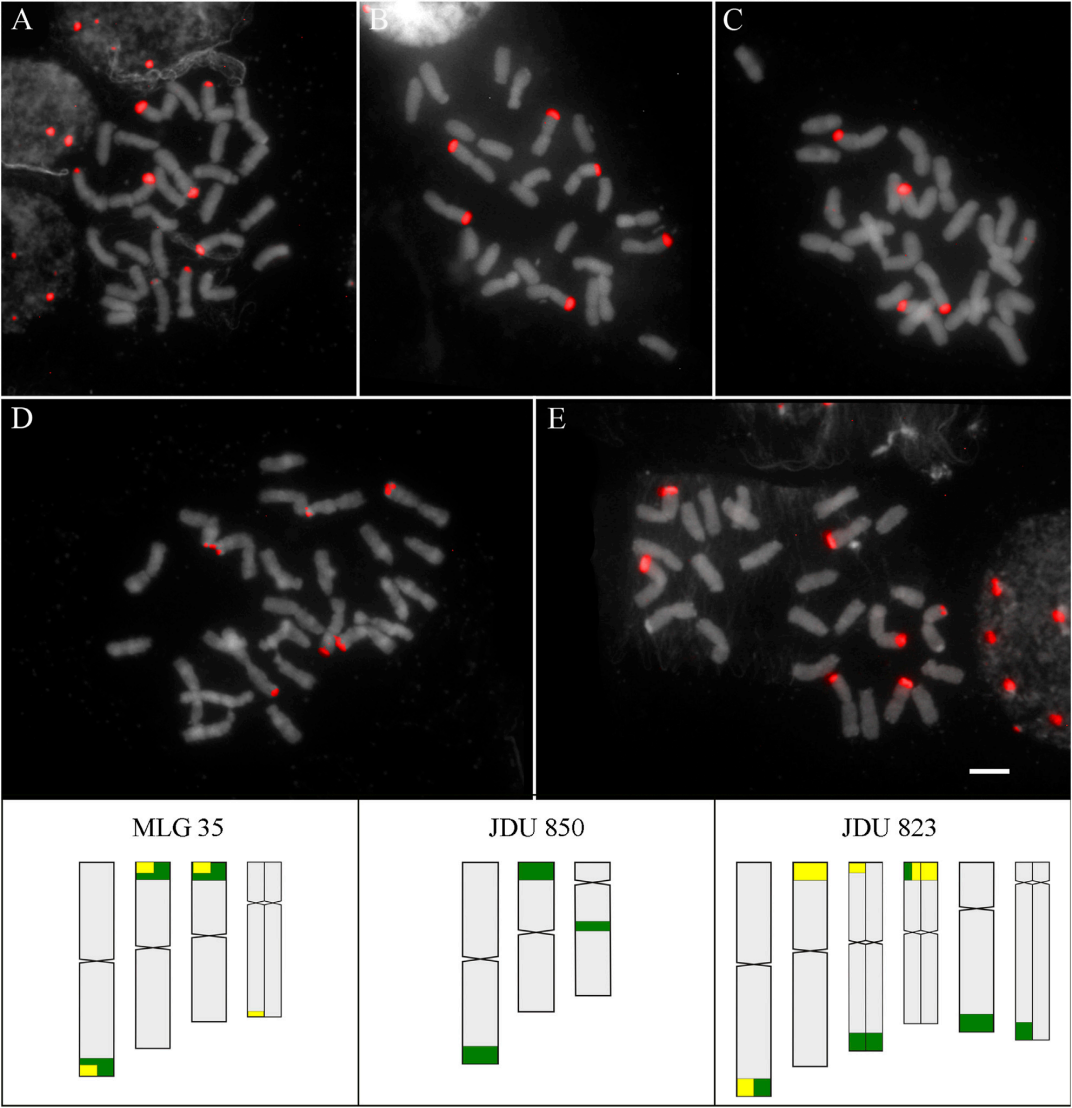
SatDNA D2 and D3 in *D. cespitosa*. Above: FISH of satDNA D2 and D3 of *D. cespitosa* **(A)** D2 of JDU 823 **(B)** D2 of MLG 35 **(C)** D2 of JDU 850 **(D)** D3 of MLG 35 **(E)** D3 of JDU 823. Scale: 5 µm. Below: Idiograms of the three localities, showing the position of satDNA D2 (green) and D3 (yellow). Heteromorphic chromosome pairs are shown divided into two.

The hybridization pattern of D2 and D3 satDNA was studied in three and two localities of *D. cespitosa*, respectively, revealing intraspecific variation. The satDNA D2 showed six loci in two localities separated by approximately 26 km (JDU 850 and MLG 35), and eight loci for the most distant locality (JDU 823). The satDNA D3 showed three loci for both studied localities (JDU 823 and MLG 35). In addition to the numerical variation, the disposition of loci showed variation for both probes in all localities (Table 1; Figure 4).

For the reconstruction of ancestral states of satDNA, we used a total of six chromosomal traits, two discrete and four continuous (Supplementary Table 1). None of these showed a significant phylogenetic signal according to parameter λ (Pagel, 1999), despite some of them showing values of 1. For discrete traits, the analysis with ER (the better fitted model) did not reflect high probabilities on the ancestral state (presence/absence) in most of the nodes, except in common ancestors of small clades. The continuous traits fitted with a BM model, and does not suggested any trend in the number of satDNA loci in *Deschampsia*. However, increases or decreases of loci can be seen for small groups or species, as an increase of satDNA D3 in the *Deschampsia* sp/*D. cordillerarum/D. mendocina* clade and the *D. antarctica*/*D. patula* clade. Particularly, *Deschampsia* sp shows an increase in the number of signals for both satDNA D2 and D3, while *D. elongata* shows an increase in D12. The satDNA D13 was constant in terms of loci number per basic complement, with the exception of a decrease in *D. kingii*, a polyploid species (Supplementary Figure S5).

## Discussion

Constitutive heterochromatin is an important genomic component, essential in nuclear architecture, DNA repair, and genome stability (Strom et al., 2017). The first analyses of heterochromatin in *Deschampsia* were made on *D. cespitosa* from Europe, by the use of the C-band technique, which detected big heterochromatin blocks at subterminal regions of most chromosomes and at intercalary regions of a few chromosomes (Garcia-Suarez et al., 1997), a frequent feature of several genera of tribe Poeae. Heterochromatin is commonly composed of several kinds of tandem repetitive sequences (satDNA), usually with unknown origin and function, considered as fast-changing genomic components (Fuchs et al., 1994; Kuipers et al., 2002; Winterfeld and Röser, 2007a). The variation of such repetitive DNA between related species could provide information about its evolution, and clarify the phylogenetic relationships between lineages (Beckmann and Soller, 1986; King et al., 1995). Highly repetitive sequences were detected for the first time in *D. cespitosa* by membrane hybridization of four satDNA previously isolated from *Helictotrichon* (which belong to the same tribe, Poeae) and were reported in several other grasses (CON1, CON2, COM1, and COM2) (Grebenstein et al., 1995, 1996). Further studies of the satellitome corroborated the presence of these satDNAs in South American *D. cespitosa* and *D. antarctica* (except for COM2), as well as described another 31 satDNA families, with two kinds of hybridization patterns: “mainly clustered”, and mixed “clustered and dispersed” (González et al., 2018, 2020). The satDNA here studied correspond with the pool of clustered satDNA, of which D2 and D3 are the most abundant satDNA of *D. antarctica* and *D. cespitosa* genomes, while D12 and D13 (homologous with CON1 and CON2, respectively) are commonly detected in the genome of several Poaceae (Alix et al., 1998; Röser et al., 2014; Zhao et al., 2017).

As originally described, the species of *Deschampsia* are characterized by their appearance in subterminal regions of AT-rich heterochromatin (as observed with DAPI staining post-FISH), and these regions also show several kinds of clustered satDNA as we found here for D2, D3, and D12 (Garcia-Suarez et al., 1997; Winterfeld and Röser, 2007a; Amosova et al., 2017; González et al., 2020, 2021). The presence of AT-rich repetitive sequences has been frequently detected at the terminal region of other grass genomes (Flavell, 1986; Kubis et al., 1998; Winterfeld and Röser, 2007a; Hemleben et al., 2007), and such repetitive DNA accumulation may be caused by the low recombination rate in the telomeric chromosome regions (Charlesworth et al., 1994). On the other hand, intercalary heterochromatic bands are less common in *Deschampsia* chromosomes, but they may also coincide with satDNA. Here we found intercalary distribution for D13 and D2 in acrocentric chromosomes only, however, previous studies showed that chromosome intercalary regions in *Deschampsia* are also rich in dispersed satDNA (González et al., 2020).

The variability of loci number and chromosome distribution of repetitive DNA between and within species is common for *Deschampsia*. Studies of rDNA distribution (*18-5.8-26S* and *5S*) have shown chromosomal variation related with phylogeny of species and geographic distances (González et al., 2016, 2021). Here we found high variation of the distribution pattern of satDNA D2, D3, and D12 between *Deschampsia* species. Particularly, D13 was the only satDNA which did not show any variation in its distribution between the studied species. Even *D. kingii*, which is tetraploid with 52 chromosomes, showed only one locus of D13 in the same position and chromosome type as the rest of the species. Previous observations between *D. antarctica* and *D. cespitosa* showed the same hybridization patterns only for two of the thirteen studied satDNA families, D13 and D14 (homologous to satDNA pSc200, widely spread in tribes Poeae and Triticeae), which indicates that this conservation of the distribution pattern is not the most frequently observed in satDNA families (Bedbrook et al., 1980; González et al., 2020). Despite the fact that satDNA could enhance the phylogenetic information of species (Plohl et al., 2012), we found a lack of association between phylogenetic relationships of *Deschampsia* and the chromosomal distribution and the loci number of satDNA. The genomic abundance of satDNA changes widely and rapidly between generations, leading to polymorphism of arrays (Plohl et al., 2012), and therefore some variation between and within species may be expected.

Here we report different hybridization patterns of D2 and D3 between localities of *D. cespitosa* from South America. The intraspecific variation of chromosomal distribution of repetitive DNA has been previously found for *Deschampsia*, by studying the rDNA and satDNA in several localities of *D. antarctica,* and rDNA in localities of *D. cespitosa* (Winterfeld and Röser, 2007b; González et al., 2016, 2018, 2021; Navrotska et al., 2018). In both cases, this variability seems to be related with geographic distances since the nearest localities share more loci with each other, and satDNA showed more variation than rDNA. On the other hand, the Patagonian localities of other species, such as *D. elongata* and *D. parvula* also showed different hybridization patterns of rDNA than localities from other world regions (United States and Falkland Islands, respectively) (Amosova et al., 2017, 2021).

**FIGURE 5:**
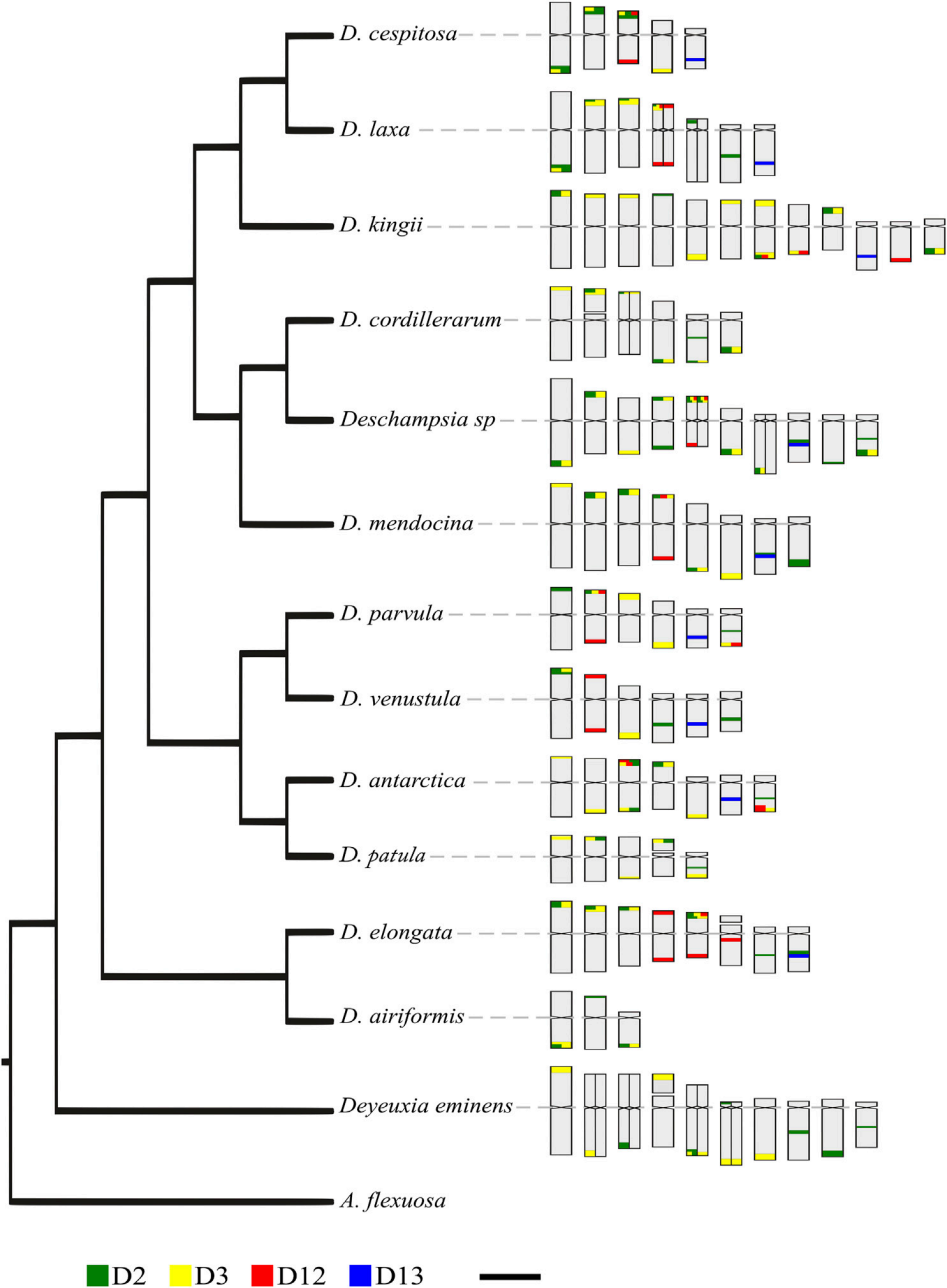
Phylogenetic relationships of studied species and idiograms showing the chromosome position of satDNA. The phylogeny is based on González et al. (2021), trimming the taxa is not studied here. Heteromorphic chromosome pairs are shown divided into two. Scale: 5 µm.

The intraspecific chromosomal variability of satDNA could explain the lack of association between chromosomal changes and phylogenetic relationships observed in *Deschampsia* species (Figure 5). Since the chromosome morphologies and karyotype composition remain highly conserved in *Deschampsia* (Kawano, 1963; Albers, 1980; Winterfeld and Röser, 2007b; Cardone et al., 2009; González et al., 2016, 2021), the high variability of sequences distribution is likely a consequence of differential amplification and loss of repetitive DNA loci between lineages (Fry and Salser, 1977; Ruiz-Ruano et al., 2016). Also small rearrangements can be involved, without notable changes in chromosome numbers and morphologies (Raskina et al., 2008; Nani et al., 2016). The divergent evolution of satDNA may be involved in micro and macroevolutionary processes, facilitating the reproductive isolation of groups by the emergence of chromosomal barriers, eventually giving rise to speciation (Noor et al., 2001; Widmer et al., 2009; Plohl et al., 2012). At the same time, the eventual secondary contacts of lineages with a differentiated pattern of repetitive DNA could give rise to allopolyploids (Mallet, 2007; Soltis and Soltis, 2009; González et al., 2016).

Despite the fact that most repetitive DNA are considered as fast evolutionary elements, mainly in abundance but also in sequence, some satDNA may persist over long evolutionary periods (Plohl, 2010). This may be the case of several satDNAs found in *Deschampsia* which show homology with sequences previously described in Poaceae (Röser et al., 2014; González et al., 2020). The homology of D12 and D13, with the previously described CON1 and CON2 was of 74 and 78% (González et al., 2018). The repetitive DNA shared between distant Poaceae tribes or genus may be related with an ancestral repetitive DNA pool in grasses which evolved differentially between lineages (Hemleben et al., 2007). This is known as the “library model”, and implies that the satDNA profile of a species is the result of differential amplification or contraction from a sequences set of an ancestral genome (Fry and Salser, 1977; Plohl et al., 2012). Some satDNAs may persist in genomes as low copy number sequences over long evolutionary periods and eventually amplify giving rise to high repetitive DNA (Ugarkovic, 2008; Röser et al., 2014). Other satDNA can be lost in some lineages as a consequence of an unequal exchange, which would eventually cause a single copy sequence and then be lost by drift (Charlesworth et al., 1986). One possible reason for low evolutionary rates is the preference of some monomers over others due to their potential function (Plohl, 2010; Plohl et al., 2012). Also, the heterochromatic environment can cause an extreme conservation of the sequence due to low recombination frequency, which together with the concerted evolution would cause a prolonged persistence of repetitive sequences throughout evolutionary time (Charlesworth et al., 1986).

The satDNA D12 (CON1) and D13 (CON2) could have had an early appearance in Poaceae; specifically, D13 seems to belong exclusively to tribe Poeae, while D12 is widespread in Poaceae including members of subfamilies Pooideae, Panicoideae, Chloridideae, and Oryzoideae (Grebenstein et al., 1996; Alix et al., 1998; Winterfeld and Röser, 2007a; Röser et al., 2014; Zhao et al., 2017). The other two satDNA here studied, D2 and D3, seem to be exclusively of *Deschampsia* lineage (González et al., 2020). Since we found D2 and D3 in all studied *Deschampsia* and *Deyeuxia eminens*, it is likely these satDNAs originated in a recent common ancestor between these two groups. As has been previously found, the sect. *Stylagrostis* from *Deyeuxia* (where *D. eminens* belong) seems to be highly related with *Deschampsia* (Saarela et al., 2017; González et al., 2021), and the presence of D2 and D3 in the *D. eminens* genome supports their monophyletic relationship.

Despite the chromosomal distribution variability of repetitive DNA (González et al., 2020, 2021), the presence of a clear hybridization for all probes in all *Deschampsia* species indicates likely conservation of the monomer sequence in the group. This can be a consequence of low evolutionary rates of the studied satDNA, or a recent species radiation. Either way, since satDNA are considered rapid evolution DNA, the presence of all studied satDNA in *Deschampsia* and *Deyeuxia eminens* genomes reinforce the idea of a low genetic differentiation between species (in terms of DNA sequences), as previously found in another studies of this group (Chiapella, 2007; Chiapella et al., 2011; Fasanella et al., 2017; González et al., 2020, 2021). In fact, previous studies suggest that karyotype differentiation between *Deschampsia* species is more associated with changes in chromosomal distribution than changes in the DNA sequence of satDNA (González et al., 2020; Amosova et al., 2021).

## Data Availability

The original contributions presented in the study are included in the article/Supplementary Material, further inquiries can be directed to the corresponding author.
