# Supplementary material for: Chromosomal Differentiation of Deschampsia (Poaceae) Based on Four Satellite DNA Families
Source: Front Genet. 2021 Sep 21;12:728664. doi: 10.3389/fgene.2021.728664 (PMC8490763; doi:10.3389/fgene.2021.728664)
Supplement: Supplementary file 1 [file DataSheet1.pdf]

Supplementary material

Table S1: Satellite DNA chromosomal traits used in the reconstruction of ancestral states. P: presence; A: absent, Mk: Markov model; ER: equal rates model; ARD: all rates different model; BM: Brownian motion model.

<table>
<tr><th colspan="3">Trait</th><th>Trait state</th><th>Better fitted model</th><th>Pagel´s lambda</th></tr>
<tr><td rowspan="4">Discrete</td><td rowspan="2">1</td><td rowspan="2">SatDNA D3 on st position of sm chromosomes</td><td>P</td><td rowspan="2">Mk-ER</td><td rowspan="2">0.00</td></tr>
<tr><td>A</td></tr>
<tr><td rowspan="2">2</td><td rowspan="2">SatDNAs D3and D2 on st position of sm chromosomes</td><td>P</td><td rowspan="2">Mk-ER</td><td rowspan="2">1.00</td></tr>
<tr><td>A</td></tr>
<tr><td rowspan="4">Continuous</td><td>3</td><td>Number of satDNA D2 hybridization signals per basic chromosomal complement x</td><td>-</td><td>BM</td><td>0.64</td></tr>
<tr><td>4</td><td>Number of satDNA D3 hybridization signals per basic chromosomal complement x</td><td>-</td><td>BM</td><td>0.88</td></tr>
<tr><td>5</td><td>Number of satDNA D12 hybridization signals per basic chromosomal complement x</td><td>-</td><td>BM</td><td>0.88</td></tr>
<tr><td>6</td><td>Number of satDNA D13 hybridization signals per basic chromosomal complement x</td><td>-</td><td>BM</td><td>0.00</td></tr>
</table>

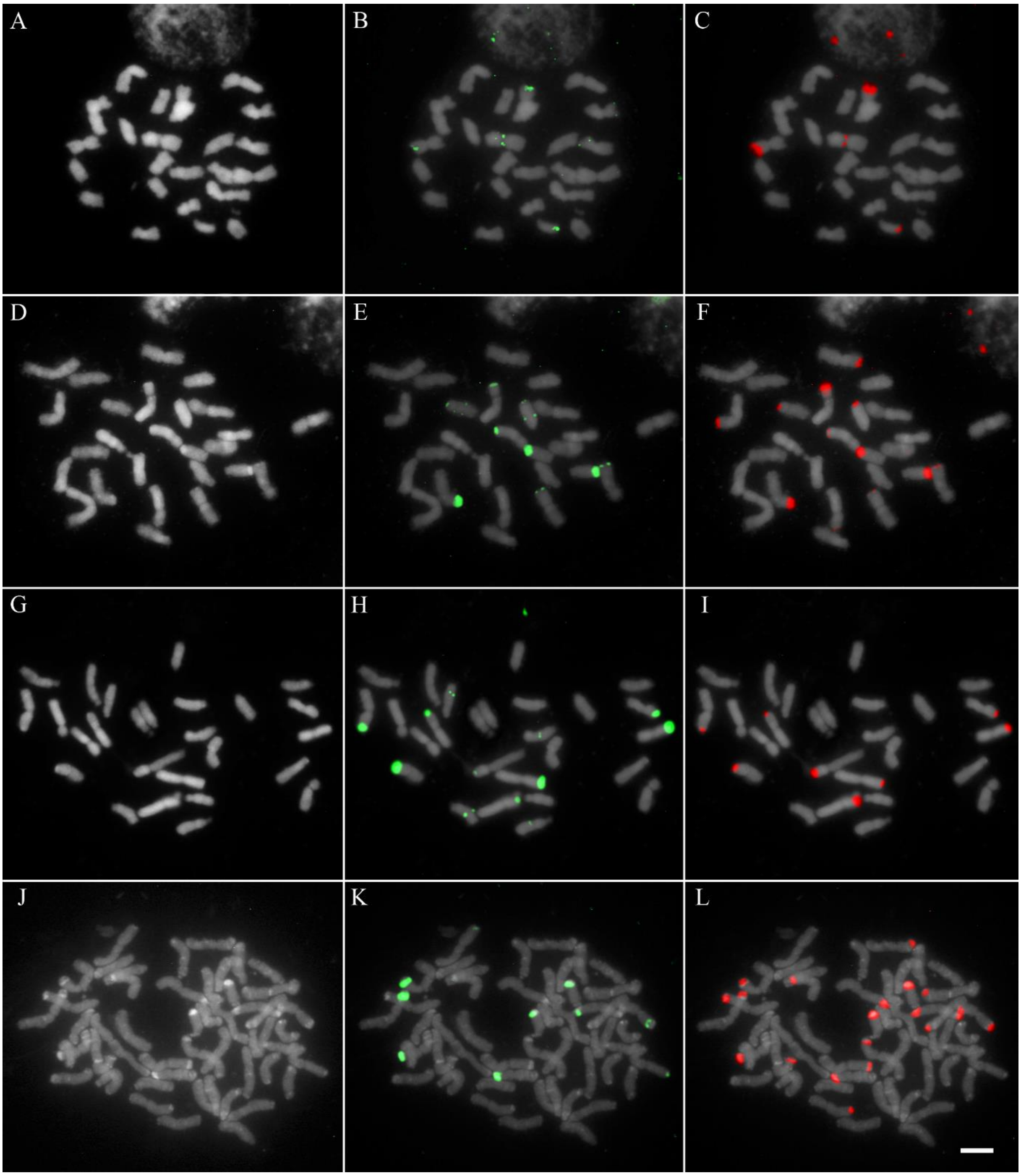

Figure S1. SatDNA FISH of the studied species. **(A)** DAPI staining of *D. airiformis*, **(B)** D2 - *D. airiformis*, **(C)** D3 - *D. airiformis*, **(D)** DAPI staining of *D. cordillerarum*, **(E)** D2 - *D. cordillerarum*, **(F)** D3 - *D. cordillerarum*, **(G)** DAPI staining of *D. elongata*, **(H)** D2 - *D. elongata*, **(I)** D3 - *D. elongata*, **(J)** DAPI staining of *D. kingii*, **(K)** D2 - *D. kingii*, **(L)** D3 - *D. kingii*. Scale: 5 μm.

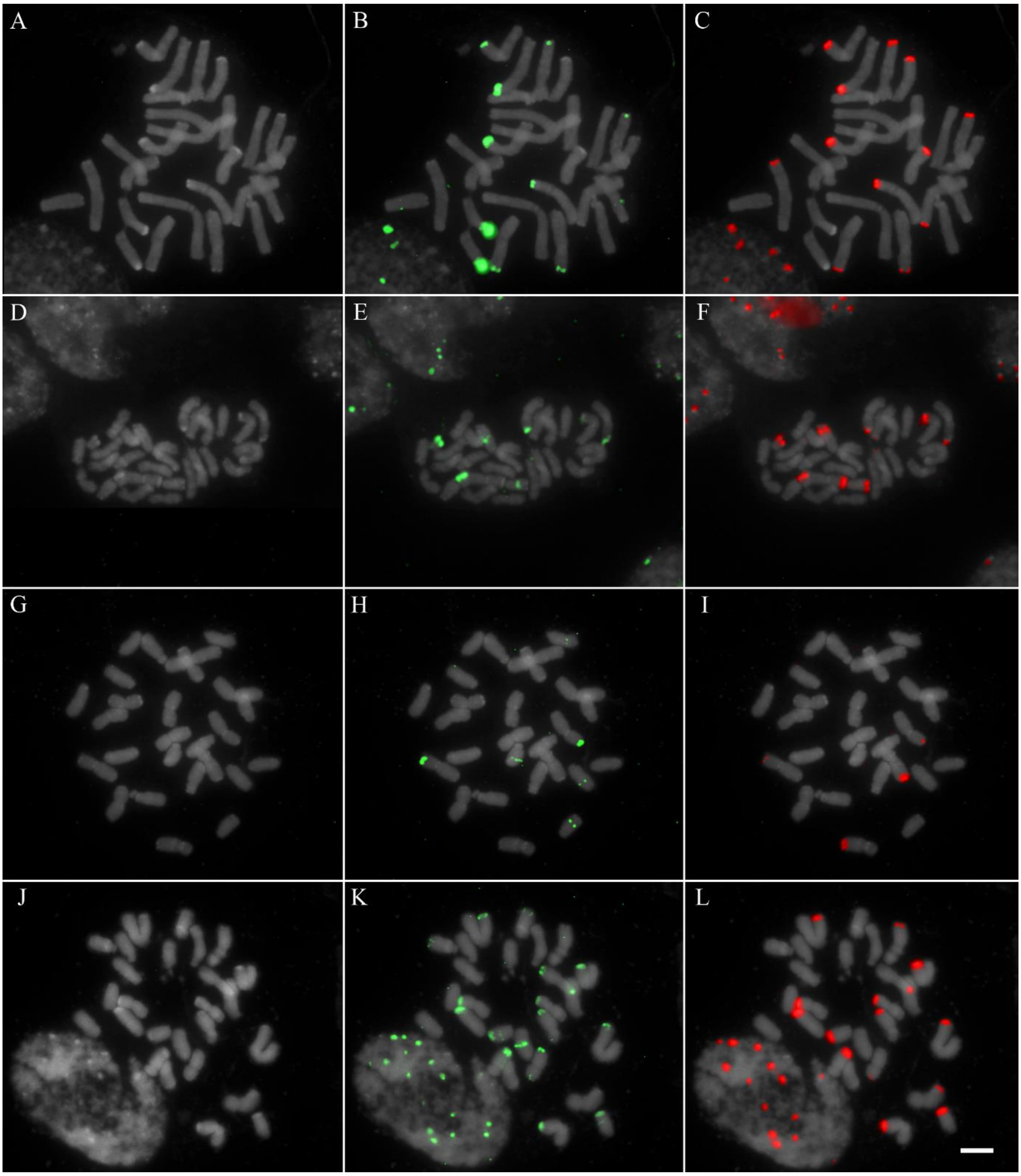

Figure S2. SatDNA FISH of the studied species. **(A)** DAPI staining of *D. mendocina*, **(B)** D2 - *D. mendocina*, **(C)** D3 - *D. mendocina*, **(D)** DAPI staining of *D. patula*, **(E)** D2 - *D. patula*, **(F)** D3 - *D. patula*, **(G)** DAPI staining of *D. venustula*, **(H)** D2 - *D. venustula*, **(I)** D3 - *D. venustula*, **(J)** DAPI staining of *Deschampsia* sp MLG 81, **(K)** D2 - *Deschampsia* sp MLG 81, **(L)** D3 - *Deschampsia* sp MLG 81. Scale: 5 µm.

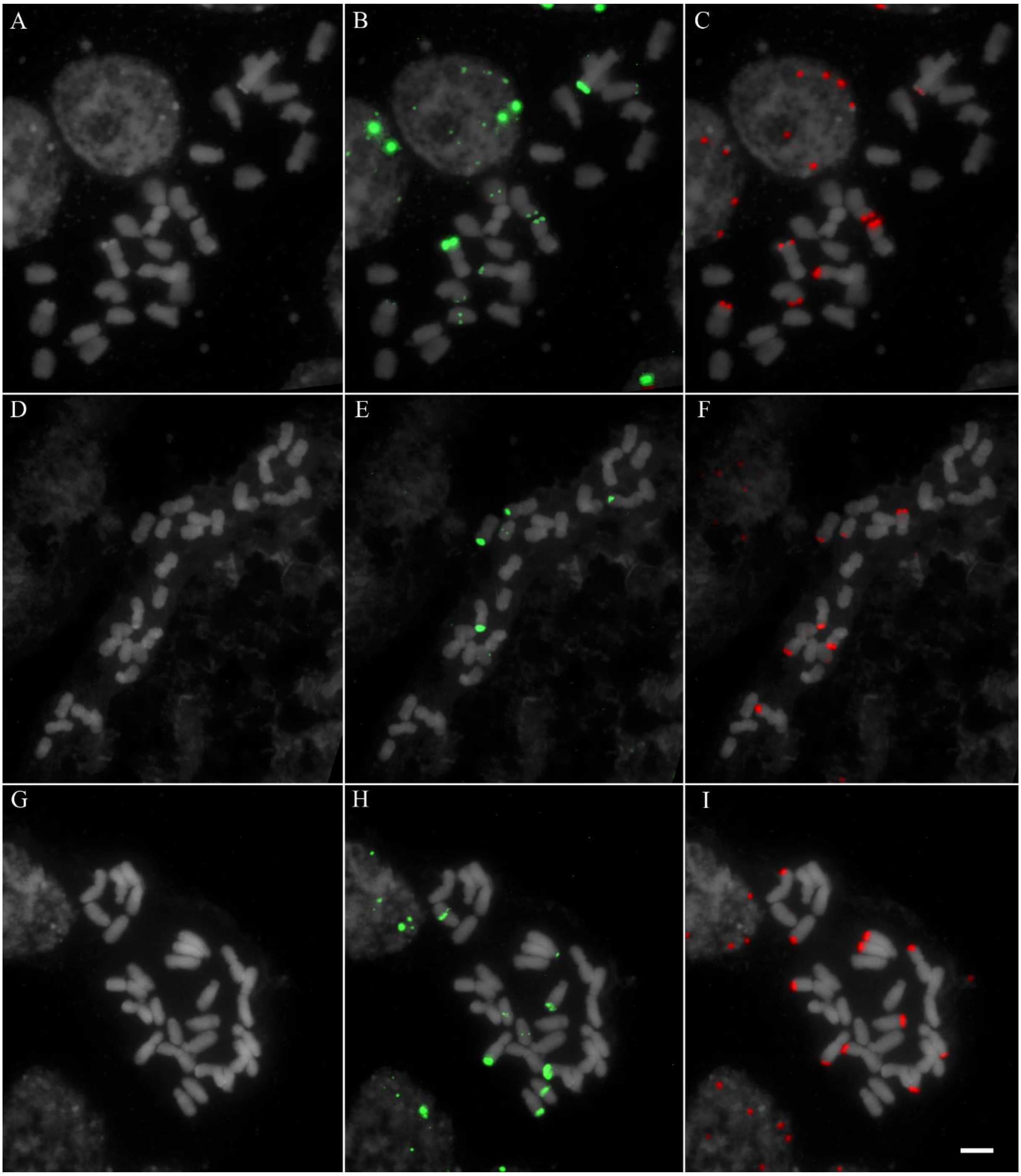

Figure S3. SatDNA FISH of the studied species. **(A)** DAPI staining of *D. laxa*, **(B)** D2 - *D. laxa*, **(C)** D3 - *D. laxa*, **(D)** DAPI staining of *D. parvula*, **(E)** D2 - *D. parvula*, **(F)** D3 - *D. parvula*, **(G)** DAPI staining of *Deyeuxia eminens*, **(H)** D2 - *Deyeuxia eminens*, **(I)** D3 - *Deyeuxia eminens*. Scale: 5 µm.

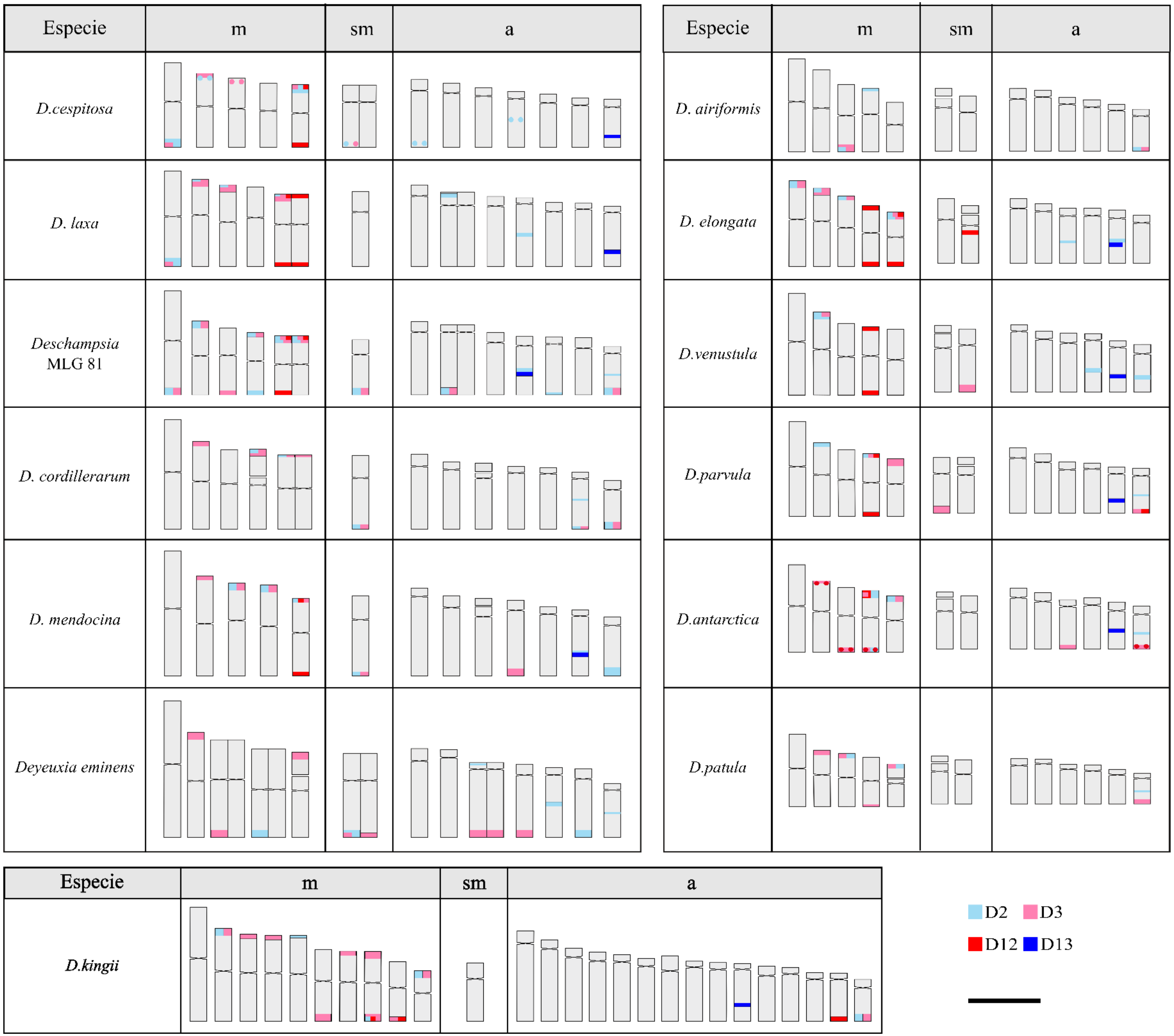

Figure S4. Idiograms of species showing the loci of the four studied satDNA families D2, D3, D12 and D13. Heteromorphic chromosome pairs for a given satDNA are drawing duplicated. Dots in *D. cespitosa* and *D. antarctica* represent variable loci, depending of the locality (Gonzalez et al. 2018). Scale: 5 µm

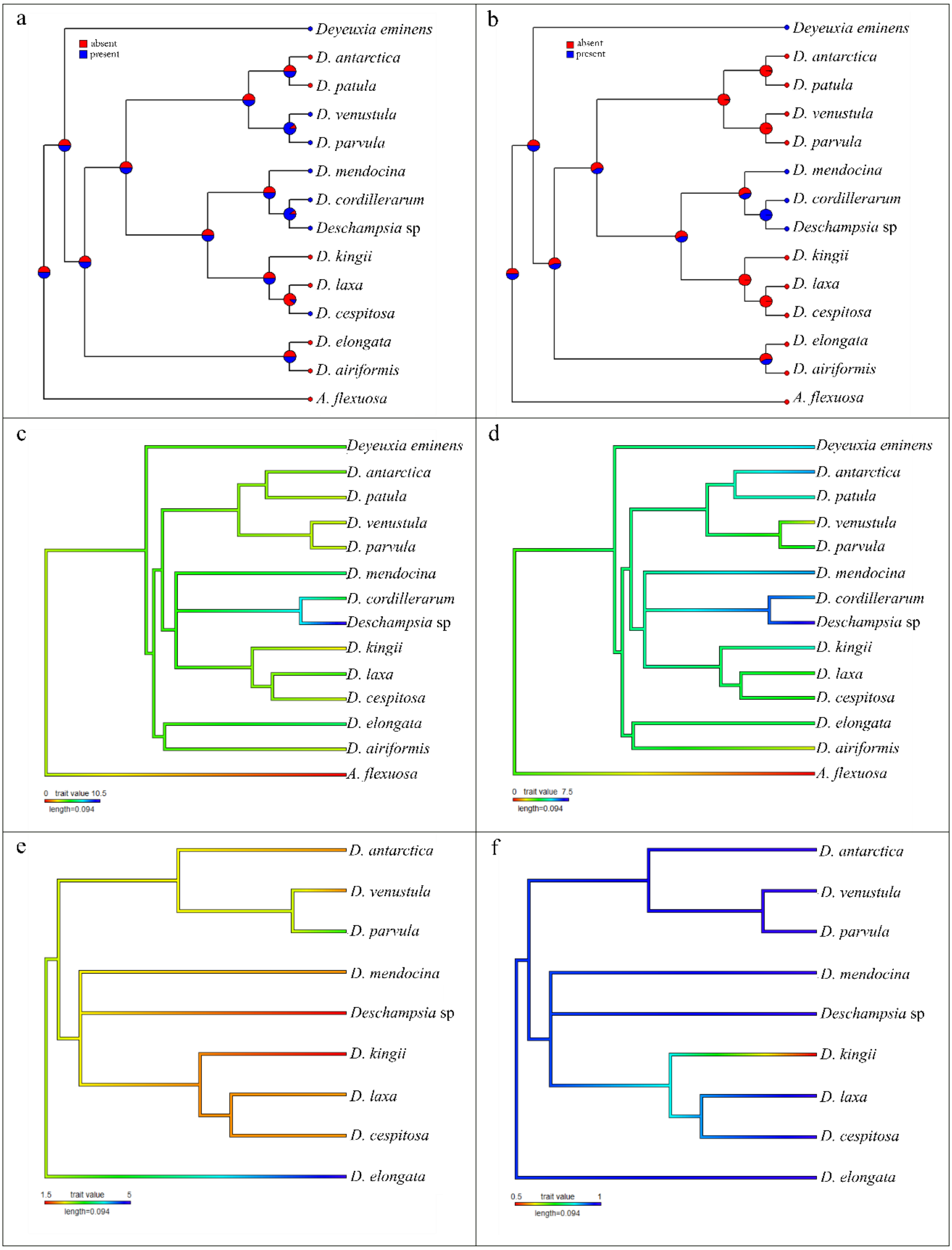

Figure S5. Reconstruction of ancestral states of satDNA traits. **a.** SatDNA D3 on st position of sm chromosomes. **b.** SatDNAs D3and D2 on st position of sm chromosomes. **c.** Number of satDNA D2 hybridization signals per basic chromosomal complement x. **d.** Number of satDNA D3 hybridization signals per basic chromosomal complement x. **e.** Number of satDNA D12 hybridization signals per basic chromosomal complement x. **f.** Number of satDNA D13 hybridization signals per basic chromosomal complement x.
